# Palindromes in DNA—A Risk for Genome Stability and Implications in Cancer

**DOI:** 10.3390/ijms22062840

**Published:** 2021-03-11

**Authors:** Marina Svetec Miklenić, Ivan Krešimir Svetec

**Affiliations:** Faculty of Food Technology and Biotechnology, University of Zagreb, Pierottijeva 6, 10000 Zagreb, Croatia; mmiklenic@pbf.hr

**Keywords:** DNA palindromes, quasipalindromes, palindromic amplification, palindrome-mediated genetic recombination, carcinogenesis

## Abstract

A palindrome in DNA consists of two closely spaced or adjacent inverted repeats. Certain palindromes have important biological functions as parts of various cis-acting elements and protein binding sites. However, many palindromes are known as fragile sites in the genome, sites prone to chromosome breakage which can lead to various genetic rearrangements or even cell death. The ability of certain palindromes to initiate genetic recombination lies in their ability to form secondary structures in DNA which can cause replication stalling and double-strand breaks. Given their recombinogenic nature, it is not surprising that palindromes in the human genome are involved in genetic rearrangements in cancer cells as well as other known recurrent translocations and deletions associated with certain syndromes in humans. Here, we bring an overview of current understanding and knowledge on molecular mechanisms of palindrome recombinogenicity and discuss possible implications of DNA palindromes in carcinogenesis. Furthermore, we overview the data on known palindromic sequences in the human genome and efforts to estimate their number and distribution, as well as underlying mechanisms of genetic rearrangements specific palindromic sequences cause.

## 1. Introduction

As the exploration of various genomes, including our own, moves forward thanks to ever more advanced and more easily available techniques, it is becoming clear that the genome is much more than the assembly of genes. The architecture of the genome is complex and wondrous, with sequences that play various important roles, but also those, such as DNA palindromes, that create fragile sites (i.e., sites prone to potentially lethal chromosome breakage), thus endangering genome stability. As a response to the breakage (double-strand break—DSB) in DNA, the cell employs various repair mechanisms which can ultimately result in genetic rearrangements. First, in Section 2 of this review, we explore the recombinogenic nature of palindromic sequences. We explain why palindromes are recombinogenic and what is the current understanding of molecular mechanisms underlying palindrome recombinogenicity in eukaryotic cells. In Section 3, we focus on the role DNA palindromes play in carcinogenesis. Palindromic amplification of genes is one of the hallmarks of cancer cells and is often linked to poor treatment prognosis. We discuss the mechanisms of de novo palindrome formation and palindromic amplification in cancer cells, as well as possible roles preexisting DNA palindromes and short inverted repeats might have in these processes. Finally, in Section 4, we overview the available data on palindrome number and distribution in eukaryotic genomes and discuss the newest experimental evidence regarding mechanisms underlying recurrent genetic rearrangements instigated by known palindromic sequences in the human genome.

## 2. The Recombinogenic Nature of Palindromic Sequences

### 2.1. DNA Palidromes Can Form Secondary Structures

**Figure 1:**
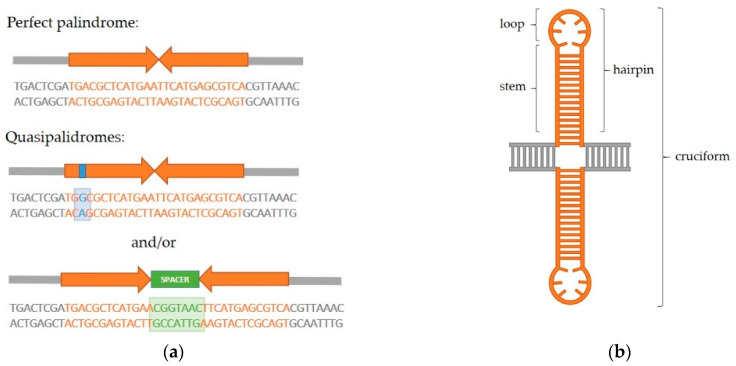
Palindromic sequences and secondary structures. (**a**) Types of palindromic sequences. A perfect palindrome consists of two identical inverted repeats adjacent to one another. A quasipalindrome can contain mismatches between the two inverted repeats and/or a central spacer region. (**b**) Palindromic sequences can form secondary structures due to intrastrand base pairing. In ssDNA, the hairpin structure, consisting of a stem and a loop, is formed. Two hairpins, one across from the other in a dsDNA, together constitute a cruciform structure.

A palindrome in DNA is a sequence consisting of two identical or highly similar inverted repeats which are either adjacent to one another or separated by a spacer region (Figure 1a). If the repeats (also called the palindrome arms) are identical and have no spacer in between, the palindrome is referred to as perfect. The term quasipalindrome can be used to refer to a non-perfect palindrome. Palindromes are found in genomes of all species investigated so far and they often play important roles as binding sites for homodimeric proteins, parts of promoters, replication origins or other regulatory sequences [1]. However, many of the discovered palindromes have no known biological function and can be relatively long (from several dozen to several hundred base pairs). If a palindrome is of sufficient length, intrastrand base pairing can occur and this results in formation of secondary structures in DNA (Figure 1b). In the single-stranded DNA, a hairpin structure forms, while in the double-stranded DNA, a cruciform structure consisting of two hairpins, one in each strand, forms. Each hairpin consists of a stem comprised of complementary paired inverted repeats and a loop. The loop either consists of bases within the spacer, if such region is present in a specific palindrome, or of four–six bases which lie in the center of symmetry of the two inverted repeats and cannot be complementarily paired due to the rigidity of the DNA strand [2,3]. It is considered that a hairpin structure can occur in the single-stranded lagging strand during DNA replication. A cruciform structure forms in dsDNA by gradual extrusion which begins at the center of the palindrome. After denaturation of a short region of DNA at the center, intrastrand base pairing occurs and a small proto-cruciform forms which can further extrude into a larger cruciform [2,4,5]. Conditions which lead to cruciform extrusion have been extensively studied in vitro [6,7,8,9] and theoretical kinetic models of cruciform extrusion were postulated [10,11]. These experiments show that cruciform extrusion is thermodynamically unfavorable, and in those early studies, it was debated if extrusion is even possible in vivo. However, processes such as transcription and replication can increase the density of negative supercoils in DNA. The added torsion can be released through cruciform extrusion and this is considered to be a mechanism driving extrusion in vivo [12,13,14].

Palindromes which have the potential to form secondary structures in DNA present a risk for genome stability since these structures can result in double-strand DNA breaks (DSBs) which in turn lead to genetic recombination, potentially resulting in translocations, deletions or gene amplifications with various consequences for the cell and organism. A specific palindrome is more likely to lead to DSB and subsequent recombination (i.e., is more recombinogenic) if the likelihood for the secondary structure formation is higher and if the structure is more stable once it forms. Thus, palindromes consisting of longer inverted repeats sharing a higher degree of sequence similarity and having a shorter or absent spacer present a higher risk for the genome stability. These principles have not only been experimentally proven for palindromes in model organisms [5,15,16,17,18,19,20] but also noted for palindromes with varying spacer length and arm length polymorphisms within the human population [21].

### 2.2. Molecular Mechanisms of Palindrome Recombinogenicity

**Figure 2:**
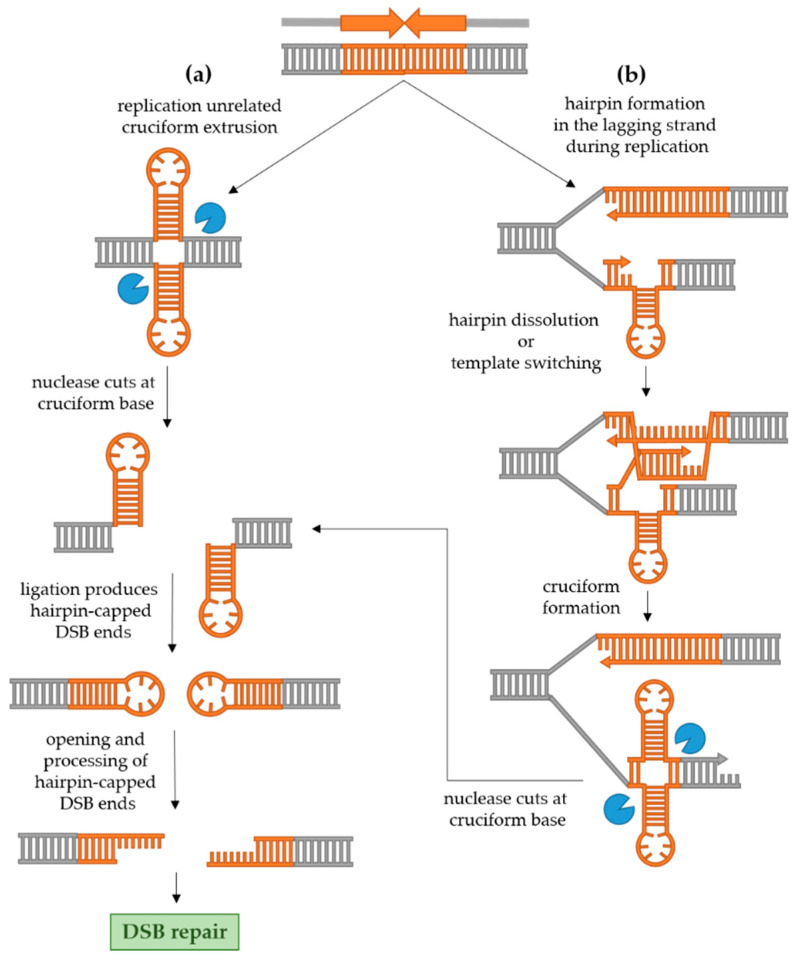
Mechanisms of palindrome recombinogenicity. (**a**) Independently from DNA replication, a cruciform structure can extrude in the dsDNA. The cruciform is cut by a structure-specific endonuclease, after which ligation produces closed hairpin-capped double-strand break (DSB) ends. Such ends must be opened and processed into conventional DSB ends which can undergo repair. (**b**) During replication, especially if the replication machinery is impaired, a hairpin can form in the ssDNA of the lagging strand, causing replication stalling. The hairpin can be dissolved by helicase, or, alternatively, template switching allows synthesis of the nascent strand using the leading strand as a template. This produces a nascent complementary strand containing a hairpin as well, across the hairpin in the lagging strand. Again, the resulting cruciform can be cut by a structure-specific endonuclease, followed by formation of hairpin-capped DSB ends, their processing and subsequent DSB repair.

Molecular mechanisms of palindrome recombinogenicity have been investigated in model organisms *Escherichia coli* and *Saccharomyces cerevisiae* as well as in human cells. While in *E. coli* the recombinogenicity of palindromes seems to be exclusively replication-related [22,23,24], in eukaryotic cells, both hairpin formation during replication and cruciform formation not related to replication seem to be at play [25,26,27]. The mechanisms of DSB formation and processing of palindromic sequences in eukaryotic cells are shown in Figure 2 and explained in detail in Section 2.2.1 and Section 2.2.2.

#### 2.2.1. Replication-Independent Palindrome Recombinogenicity

The cruciform structure in dsDNA can be cleaved at the base by a structure-specific endonuclease. Since the base of the cruciform resembles a Holliday junction, the likely candidates for cruciform cleavage are resolvases. This idea is in accordance with the fact that the cruciform is efficiently cut by bacterial resolvase RuvC as well as T4 DNA endonuclease VII in vitro [7,28]. In a culture of human cells, Inagaki et al. [29] used two non-replicating plasmids carrying two palindromes originating from the human genome (PATRRs, palindromic AT-rich repeats) to demonstrate that the cruciform structure is recognized and cut exclusively by Gen1 resolvase, and not by Mus81-Eme1 or Slx1–Slx4. In yeast, however, the cruciform-cutting endonuclease remains elusive [5]. Experimental evidence suggests that the two nicks created by the action of resolvase are usually ligated in vivo, since replication of DNA with an unprocessed hairpin-capped end leads to large palindromic duplication [25,30]. Such ends need to be processed into conventional DSB in order to be repaired. In human cells, the hairpin-capped DSB ends are opened by Artemis [29], while in yeast, the Sae2 endonuclease together with the MRX complex processes the ends [25]. Once hairpin-capped ends are processed, the conventional DSB can undergo repair. In yeast, the predominant mechanism of DSB repair is homologous recombination, while in human cells, the break will most often be repaired by non-homologous end joining (NHEJ) [31].

#### 2.2.2. Replication-Dependent Palindrome Recombinogenicity

During DNA replication, the palindromic sequence can result in the formation of a hairpin in the single-stranded lagging strand which can cause replication stalling and a double-strand break. The stalling of the replication fork at a palindrome consisting of two inverted *Alu* repeats separated by a 12 bp spacer was demonstrated in *E. coli*, yeast and primate fibroblasts in the work by Voinegau et al. [26]. Interestingly, when the same repeats were separated by a longer 52 bp spacer, frequent stalling was observed only in *E. coli*, but not in eukaryotic cells. This is in line with the idea that replication stalling occurs because a hairpin is formed in a single-stranded region of the lagging strand. Namely, in prokaryotes, the Okazaki fragment initiation zone is at least 1500 bp long, while in eukaryotes, it is limited to 100–200 bp [32]. Therefore, an increase in spacer length reduces the likelihood that a central region of a palindrome which can form a hairpin will be contained within the ssDNA. However, in case DNA replication is in some way impaired (e.g., slowed because of deficiency in one of the proteins of the replication machinery), the ssDNA could potentially become abnormally long which can result in otherwise stable repeats to become unstable and recombinogenic [19,27,33]. For example, the fragile site 2 in yeast which consists of two large inverted repeats (Ty elements, each 6 kb long) separated by a large 280 bp spacer is completely stable in wild-type yeast but becomes prone to DSB and genetic rearrangements when expression of DNA polymerase α is reduced [33].

Zhang et al. [27] also demonstrated that the frequency of DSB and recombination instigated by a palindrome introduced in the yeast genome is increased in strains which lack or have a transient reduction in expression of proteins involved in DNA replication (including Pol2 and Pol3 which are catalytic subunits of DNA polymerases ε and δ, respectively, and others such as endonuclease Rad27), proteins involved in cell cycle arrest during replication, maintaining telomere stability, and proteins belonging to the Sgs1-Top3-Rmi1 complex. Interestingly, when the ends of a DSB instigated by a palindromic sequence in cells with reduced quantities of Pol2 or Pol3 were analyzed, it was found that they are also hairpin-capped, just as in the case of the cruciform resolution described above. Moreover, in this case, the MRX-Sae2 complex was also required for opening and processing hairpin-capped ends into a canonical DSB which could undergo repair. Based on these results, the authors proposed a model shown in Figure 2b. They postulated that in conditions of a slower replication, a hairpin can form in the lagging strand which can be dissolved by the Sgs1-Top3-Rmi1 complex. If the hairpin is not dissolved, a Rad51-mediated template switching occurs and the 3′ end of the nascent strand is elongated using the leading strand as a template for synthesis, which results in formation of a cruciform. This proposal is supported by the fact that in rad51 and rad54 mutants (proteins important for 3′ end invasion and pairing with the homologous sequence in conditions of slowed replication), the frequency of DSB and the recombination rate are again reduced. The cruciform is then recognized and cut by a putative endonuclease in yeast and hairpin-capped ends of a DSB are produced as described above.

## 3. DNA Palindromes and Genetic Instability in Cancer Cells

### 3.1. Palindromic Amplifications in Cancer

Genetic instability resulting in various DNA rearrangements is one of the hallmarks of cancer cells. It is not uncommon for cancer cells to feature amplifications of genes or large genomic regions. This results in an altered level of gene expression, often with an adverse effect on the disease outcome since such tumors can become more aggressive and more proliferative (e.g., amplification of HER2 oncogene in breast cancer, [34]) or resistant to drugs (e.g., amplification of TYMS gene conferring resistance to 5-flourouracil in colorectal cancer, [35]). Multiple studies [36,37] demonstrated a link between a specific pattern of amplified regions in the cancer cell genome and a certain cancer pathophysiology. For example, in their study in breast cancer samples, Hicks et al. [37] identified a specific amplification pattern confined to a single chromosome arm. This pattern consists of multiple closely spaced amplified regions (amplicons which they named “firestorms”), often with deletions in the regions between them. Hence, copy numbers of some genes are increased, while other genes are deleted, resulting in loss of heterozygosity. They showed that even a single region featuring the “firestorm” pattern in the breast cancer genome that is otherwise relatively quiet is linked to poor prognosis. Interestingly, such studies often identify recurrent amplifications (i.e., amplifications encompassing same genomic regions found in different patients) which can help to better predict disease progression and outcome and guide treatment decisions in clinics. Growing effort is being invested into understanding cancer genomics. The Cancer Genome Atlas (TCGA) program [38] has so far molecularly characterized over 20,000 primary cancers and matched normal samples across 33 cancer types. The program uses microarrays for determining copy number variations, methylation and protein expression as well as high-throughput sequencing for characterization of DNA and RNA. Amplified genomic regions (cytobands) are determined for various cancer types. Tanaka and Watanabe [39] summarized data from TCGA on recurrently amplified regions from six epithelial tumor types (breast, colorectal, lung squamous, lung adenocarcinoma, prostate and ovarian). They found, just to outline one example, that an 800-kb-long region, 8q24.21, which carries the *MYC* oncogene is amplified across all these tumors. On the other hand, tumor type-specific amplified regions were also found. They concluded that tumor type-specific amplified regions often carry lineage-specific transcription factors, while regions amplified across several cancer types often carry genes involved in fundamental processes responsible for cell proliferation. Undoubtedly, these recurrent events are, on the one hand, under a selective pressure since deletion or overexpression of certain genes (“driver genes”) can confer advantage to a tumor cell. On the other hand, however, it is likely that certain regions of the genome are predisposed to such rearrangements due to the presence of specific DNA sequences, such as palindromes.

#### 3.1.1. Palindromic Amplification in Cancer Can Arise as a Result of Iterative Break-Fusion-Bridge Cycles

**Figure 3:**
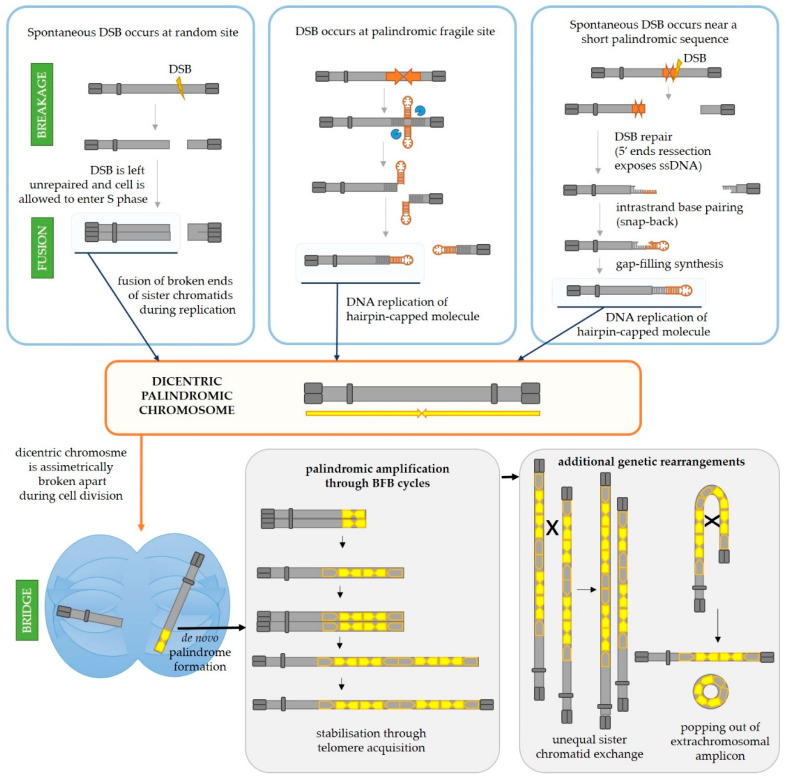
Mechanisms responsible for initiation of de novo palindrome formation followed by subsequent palindromic amplifications and additional genetic rearrangements typical for cancer cells. On the left upper panel, the initial steps of the breakage-fusion-bridge (BFB) mechanism are shown. If a DSB is left unrepaired and the DNA is subsequently replicated, the broken ends of sister chromatids can fuse to form two palindromic chromosomes, one of which will be dicentric. Middle and right upper panels depict alternative mechanisms for formation of a palindromic dicentric chromosome, an initiating event for palindromic amplification. In the middle panel, a DSB is mediated by a secondary structure-forming palindrome in the genome. As explained in detail in Section 2, this results in hairpin-capped DSB ends. If left unprocessed, the replication of the centromere-proximal hairpin-capped molecule results in an dicentric chromosome. On the upper right panel, the fold-back priming mechanism is depicted. If a DSB occurs in the vicinity of a palindrome or short inverted repeats, the DSB repair, which normally involves 5′ end resection, will expose the repeats in the ssDNA form. This can allow for intrastrand base pairing (fold-back) and the folded 3′-end can then serve as a primer for gap-filling DNA synthesis. The resulting hairpin-capped molecule can also lead to occurrence of a dicentric chromosome upon DNA replication. Regardless of the initial mechanism of formation, the palindromic dicentric chromosome can be asymmetrically broken during cell division and one of the resulting chromosomes will carry a de novo formed palindrome (palindromic duplication). Additional BFB cycles can result in palindromic amplifications of certain genomic regions and such chromosome could at some point become stabilized by telomere addition, thus breaking iterations of BFB cycles (lower middle panel). However, additional genetic rearrangements and gene copy number gains or losses can arise through unequal sister chromatid exchange and intrachromosomal recombination between the acquired repeats which can result in the popping out of an extrachromosomal amplicon (double minute chromosome), also a typical feature of cancer cells.

The pattern of DNA amplification characterized by multiple closely spaced amplified regions can be explained by breakage-fusion-bridge (BFB) cycles, first discovered in maize by Barbara McClintok [39,40,41,42,43] and illustrated in Figure 3. Tumor cells often carry deficiencies in one or more tumor suppressor genes important for DNA repair and DNA damage sensing as well as deficiencies in checkpoint systems which should lead the cell into cell cycle arrest and apoptosis if DNA damage or mitotic dysfunction exists [44]. Hence, if the cell is impaired in one or several of these vital processes, a double-strand break could be left unrepaired and the cell could be allowed to progress into the S phase of the cell cycle. At that point, the two segments of a broken chromosome are replicated, and the resulting four DNA strands can either fuse correctly to produce two identical chromosomes or incorrectly, thus producing one null-centric and one dicentric palindromic chromosome (Figure 3, central panel). During the cell division, the dicentric chromosome will be broken by the forces of the mitotic spindle pulling centromeres in the opposite directions. The anaphase bridges are often observed in tumor cells undergoing genetic rearrangements [45]. Each daughter cell will inherit a segment of the dicentric chromosome, but one of them will most likely inherit a somewhat larger segment than the other (depending on the position of the chromosome break during cell division) which will thus have a palindrome at its end. Such broken chromosomes are then a substrate for the next BFB cycle in daughter cells. This can go on for many cycles, ultimately resulting in accumulation of multiple palindromic gene amplifications at breakpoints. Such chromosome might finally be stabilized by telomere addition [46], but processes such as intrachromosomal homologous recombination between the acquired repeats and unequal sister chromatid exchange could drive further rearrangements and thus gene copy number gains or reductions [46,47,48,49]. Moreover, intrachromosomal recombination between the acquired DNA repeats can easily result in the popping-out of an extrachromosomal circular amplicon (named double minute chromosomes, DMs), a type of amplification event that is commonly detected in cancer cells but almost never in normal cells [50,51]. It is often the case that such DMs harbor oncogenes which are amplified either solely on the DM or both on the DM and on the chromosome. Mathematical modeling indicates that the oncogene copy number and genetic heterogeneity would be increased more effectively on DMs than chromosomal amplification, and this might contribute to accelerated evolution in cancer [52]. Hence, it is not surprising that the existence of DMs is also linked to poor prognosis and shorter survival for patients with various cancer types [51]. Additionally, certain complex DMs carrying DNA from various distant loci can arise during a process of chromothripsis (greek *thripsis*—breaking into pieces)—a single catastrophic event in which a chromosome arm is fragmented into pieces which are subsequently randomly reconnected [53].

#### 3.1.2. Relatively Short Palindromes in the Genome Can Facilitate the Initiating Event of Palindromic Amplification through the Fold-Back Priming Mechanism

Furthermore, the initiation of palindromic gene amplifications in cancer cells can also be explained by the fold-back priming mechanism (Figure 3, right panel). Namely, if a chromosome break occurs in the vicinity of an existing palindromic sequence in the genome, the processing of broken ends by DNA repair machinery (5′ end resection, [54]) will expose the palindromic sequence in a single-stranded form. This will result in a fold-back, i.e., intrastrand base pairing and hairpin formation. The 3′-end can then serve as a primer for gap-filling DNA synthesis which will result in a sealed hairpin-capped DNA. If left unopened and unprocessed (by MRX-Sae2 in yeast or Artemis in human cells), the replication of such chromosome will result in a large palindromic duplication (palindromic chromosome). It has been shown in yeast [55,56,57] and mammalian cells [58] that palindromic duplication can be mediated by relatively short inverted repeats which is consistent with this model. Butler et al. [55] demonstrated that induction of DSB by HO endonuclease on a yeast minichromosome next to a short 42 bp inverted repeat efficiently produced a palindromic chromosome. A similar result was obtained in Chinese hamster ovary cells [58] by induction of DSB with I-SceI endonuclease next to a 79 or 229 bp inverted repeat. Furthermore, by analyzing breakpoints at the center of palindromic duplication, Deng et al. [59] demonstrated that inverted repeats as short as 5–9 bp separated by 3–12 bp spacer sequences can drive large palindromic duplications in Mre11- or Sae2-deficinet yeast cells. Their results also indicate that a large proportion of snap-back events are prevented or removed by RPA (Replication Protein A), the ssDNA-binding protein which normally prevents annealing of very short homologous sequences as well as removing secondary structures from ssDNA [60]. The deficiency in RPA in *mre11* or *sae2* mutants increases the frequency of palindromic duplications by 1000-fold. Moreover, it appears that, besides small loop hairpins forming in close proximity (within 50 bp) to a DSB, a large loop hairpin after an extensive DSB end resection can be formed in yeast [61]. Although they are likely subsequently processed through different pathways using different enzymes, both types of hairpins can result in a gross chromosomal rearrangement and gene amplification.

#### 3.1.3. Longer Palindromes in the Genome Are Fragile Sites Which Can Lead to Palindromic Duplication

Moreover, if a palindrome present in the genome is long enough to have the potential to extrude into a cruciform structure, the resolution of the cruciform by an endonuclease also leads to the formation of hairpin-capped chromosome ends and potentially to palindromic chromosome formation (Figure 3, middle panel). This was demonstrated by Lobachev et al. [25] using two human *Alu* repeats separated by a 12 bp spacer inserted into a yeast chromosome. In the absence of Mre11, Xrs2, Rad50 (MRX complex) or Sae2, a large palindromic duplication occurs after DNA replication. Additionally, it was shown in yeast that palindromes can cause replication stalling [26], potentially leading to a double-strand break during replication [27], as depicted in Figure 2 (right). Further, if the stall persists, the replication fork can collapse and lead to a DSB or restart erroneously, leading to genetic rearrangements [62,63,64]. An interesting study identified genomic features in human and mouse genomes which highly depend on the ATR checkpoint kinase to maintain stability [65]. Namely, ATR is activated upon DNA polymerase stalling which results in stabilization of intermediates at stalled replication forks and prevents progression into the M phase. Sequences for which the stability is highly dependent on ATR, and thus most likely to stall replication, are those which can form secondary structures including some microsatellites, inverted retroelements and AT-rich palindromes and quasipalindromes.

Ultimately, regardless of the mechanism of formation, any dicentric chromosome could be broken apart by the mitotic spindle during cell division and initiate BFB cycles. A dicentric chromosome could also be produced upon telomere shortening and fusion of different chromosomes or sister chromatids with dysfunctional telomeres, as is the case in cells with deficient shelterin (protein complex responsible for telomere maintenance) lacking both p53 and retinoblastoma (RB) tumor suppressors [66,67]. Additionally, a palindromic duplication results in a new genomic fragile site since it produces a palindrome long enough to extrude into a cruciform structure or form a hairpin during DNA replication.

### 3.2. Challenges in Decyphering the Initiating Event Responsible for Palindromic Amplifications in Cancer

When it comes to various types of tumors isolated from patients, it can be difficult to pinpoint the initial mechanism which led to gene amplifications since the initial amplification is followed by subsequent rearrangements. Thus, it is difficult to determine whether the initial breakpoint and the molecular mechanism which led to subsequent rearrangements are common in a certain locus in various tumor types or not. Several interesting studies have aimed to unravel the origins of gene amplifications in human tumor samples. Tanaka et al. [68] developed a microarray-based approach for genome-wide analysis of palindrome formation (GAPF). Briefly, total genomic DNA from the sample is first digested with a restriction enzyme and then heat denaturated. The renaturation is performed under conditions (rapid cooling, presence of NaCl) which favor intrastrand over interstrand base pairing. Therefore, DNA palindromes quickly “snap back” and renaturate while the rest of the genome remains single-stranded. Next, the ssDNA is degraded with S1 nuclease, and the remaining dsDNA is digested with selected restriction enzymes, ligated with terminal linkers and amplified by PCR using Cy5-labelled linker-specific primers. In parallel, the DNA standard for competitive hybridization is isolated from normal human fibroblasts, subjected to the same procedure as the DNA from the tumor sample, except for the denaturation step, and labelled with Cy3. The standard and the sample DNAs are cohybridized on a human spotted cDNA microarray. The GAPF technique was later modified to reduce the detection of false positives and coupled with next-generation sequencing [69]. Using this technique to analyze samples from colorectal and breast cancer cell lines and primary medulloblastomas, the authors found [68,70] a frequent occurrence of de novo palindromes. Interestingly, in colorectal cancer cell line Colo320DM and breast cancer cell line MCF7, common loci containing DNA palindromes were detected, indicating that these regions might be susceptible to chromosomal breaks and palindrome formation. Guenthoer et al. [71] used the same GAPF technique coupled with gene copy number analysis to look into commonly amplified regions involved in breast tumorigenesis (chromosome arms 8q and 11q) and found that formation of palindromes de novo coincided with copy number gains and with amplicon breakpoints. These results point to the palindrome-associated mechanism of gene amplification. Moreover, in similar subsets of breast cancers featuring similar characteristics, similar amplified loci were found, implicating that preexisting fragile sites might initiate amplification.

Narayanan et al. [30] used yeast as a eukaryotic model organism to select and analyze gross chromosomal rearrangements resulting from hairpin-capped breaks occurring at the inverted human *Alu* repeats. The recovered rearrangements resembled those typically found in cancer cells, including palindromic gene amplifications and terminal deletions, highlighting the potential role of existing inverted repeats in the human genome in tumorigenesis. All of these studies strongly point to the conclusion that a de novo palindrome formation in cancer cells is not necessarily a random event and that, once it occurs, it serves as a platform for subsequent gene amplifications.

## 4. DNA Palindromes in the Human Genome

### 4.1. Bioinformatics in Quest for DNA Palindromes

The effort to estimate the abundance and distribution of DNA palindromes in genomes greatly relies on the use of bioinformatics tools to analyze genome sequence data. However, when reading such reports, one has to be aware of the specificities of each palindrome-counting algorithm which might result in a great number of palindromic sequences that are inherently recombinogenic to be overlooked or underrepresented. Moreover, the cloning of palindrome-containing genome fragments could be difficult or unsuccessful. In addition, some palindromes maintained on BACs (bacterial artificial chromsomes) in *E. coli* could be lost during propagation. Nonetheless, this does not diminish the value and importance of such studies. Most recently, Ganapathiraju et al. [72] constructed a catalog of human DNA palindromes and analyzed variations in these palindromic sequences in 1000 sequenced human genomes. Their algorithm starts palindrome counting by finding an 8 bp perfect palindrome and then extends the span of the sequence to each side, allowing a maximum of four mismatches between the palindrome arms. In other words, it counts only near-perfect palindromes without a spacer. Through counting, they found that the human genome contains around 13 million palindromes shorter than 40 bp, about 180,000 longer than 40 bp and 718 palindromes longer than 200 bp, with palindromes 8 and 20 bp in length being the most abundant. As shown in previous studies on the human genome [73], palindromes are mostly located in the intronic and intergenic regions, probably because extensive intrastrand base paring within mRNA molecules could have negative repercussions on processes of transcription and translation. Palindrome avoidance of coding regions was also demonstrated in yeast [74]. Strawbridge et al. [75] also found that closely spaced inverted repeats in the yeast *S. cerevisiae* genome are clustered in intergenic regions, with clustering being more pronounced in 3′ than in 5′ flanks of genes. Moreover, they noticed that repeats capable of forming cruciform structures are rare in the yeast genome. Further, in accordance with earlier studies on the human [73] genome and genomes of yeasts [74,76], the authors found long palindromes are highly AT-rich. Additionally, Ganapathiraju et al. [72] analyzed the GWAS catalog SNPs (single nucleotide polymorphisms) to see if any of the catalogued SNPs are found within palindrome sequences, possibly making them longer or more perfect. Interestingly, disease risk-associated SNPs were 14 times more likely to be found within the palindromic regions than in other regions. Furthermore, in their earlier study [77], the same research group used a similar approach to compare the 69 serum-normal and tumor genomes originating from The Cancer Genome Atlas with 1000 human genomes as a control. The aim of this study was to computationally identify differences in palindrome occurrence on chromosomes 8 and 11 between these two groups of genomes. Interestingly, they found that, in multiple cases, palindromes which happen to be near genes involved in breast carcinogenesis were among those which differed the most in cancer compared to normal cell genomes. Additionally, several other groups worked on palindrome-counting algorithms which are publicly available [78,79]. Moreover, efforts are being made to overcome other obstacles in identifying DNA palindromes in genomes. For example, it has been demonstrated that a palindromic sequence present in the human genome is much better preserved in the case the human genome fragment was cloned before sequencing in yeast *Saccharomyces cerevisiae* rather than bacteria *E. coli* [80].

### 4.2. From Short Interspersed Elements (SINEs) to Segmental Duplications—Possibilities for Palindrome Occurrence in the Human Genome

Given the great number of various repeated sequences in the human genome, it is not surprising that they can be found in various relations to one another, such as direct tandem repeats as well as closely spaced inverted repeats, i.e., palindromes. *Alu* repeats, a type of primate-specific short interspersed element (SINE), are one of the most abundant repeated elements in the human genome. They were named *Alu* because they usually feature a recognition site for the AluI restriction endonuclease [81]. They make up for about 11% of the human genome and count more than one million copies per haploid genome [82]. One *Alu* element is about 300 bp long, and based on their divergence, they are divided into subfamilies [83]. *Alu* repeats are often found at breakpoints of genetic rearrangements and are linked to genetic instability [84,85], gene copy number variations [86] and various diseases [87]. The potency of inverted *Alu* repeats to instigate palindrome-mediated genetic instability has been demonstrated by Lobacev et al. [19] in yeast. In the human genome, *Alu*-mediated rearrangements can occur as a result of recombination between various *Alu*s throughout the genome and through various molecular mechanisms (homologous recombination, single-strand annealing, template switching, etc.), and in some cases, they are likely to be palindrome-mediated. However, what portion of *Alu*-mediated rearrangements involves palindrome instability remains to be investigated. The same is true for various other repeated elements in the human genome which together constitute around 50% of the human DNA content [82].

Additionally, the human genome harbors large segmental duplications which make up for about 5% of the genome and are together clustered in about 400 regions. Segmental duplications arose during primate evolution and maintain high sequence identity between duplicated regions [88]. For example, palindromes P1–P8 on the male-specific section of chromosome Y span dozens of genes, many of which are essential for spermatogenesis. It is considered that these palindromes have an important evolutionary purpose since they allow intrachromsomal recombination in an otherwise non-recombining chromosome. Intrachromosomal gene conversion can protect against deleterious mutations, but also having an extra copy of these genes can enhance the adaptive evolution of chromosome Y [89]. However, recombination between palindrome arms can result in formation of isodicentric chromosome Y [90] and recombination between different Y-specific palindromes can result in massive deletion [91,92], all known causes of a range of sex-linked reproductive disorders, including relatively common spermatogenic failure [93]. Other segmental duplications have also been identified as fragile sites as well. Segmental duplication *KFTAP-1* on chromosome 17, which is often found as a boundary for palindromic amplification of the ERBB2 gene (amplified in up to 30% of breast tumors as well as stomach, bladder and esophageal cancers), has been shown to be susceptible to DSBs in normal human cells and even more so if the exonuclease activity of DNA repair protein Mre11 is disabled [94].

### 4.3. PATRRs and Other Known Palindromes in the Human Genome

**Figure 4:**
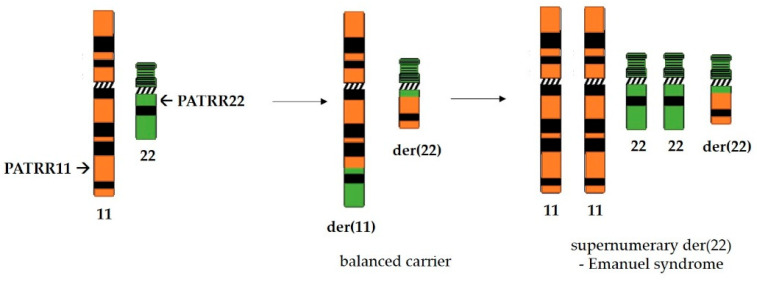
Reciprocal translocation between palindromes PATRR11 on chromosome 11 and PATRR22 on chromosome 22 in the human genome produces a generally healthy balanced carrier. However, offspring who inherit the supernumerary derivative der(22) alongside the normal chromosomal set suffer from Emanuel syndrome.

Perhaps the most thoroughly studied palindromes in the human genome are PATRRs (palindromic AT-rich repeats). These sequences are near-perfect palindromes that are quite long—several hundred base pairs with more than 90% of base pairs being AT. They do not share any significant degree of homology between them, yet, not surprisingly given their palindromic nature, they are often sites of chromosome breakage and genetic rearrangements [95]. Palindromes PATRR11 (approximately 450 bp long) and PATRR22 (approximately 595 bp long), located on chromosomes 11 and 22, respectively, are involved in the most common recurrent non-Robertsonian translocation in the human genome (Figure 4) which is the underlying cause of Emanuel syndrome [96]. Reciprocal translocation with breakpoints in these palindromic sequences produces balanced carriers which are, for the most part, healthy. They do have an increased risk of developing breast cancer [97], as well as infertility issues and recurrent pregnancy losses. However, if the small derivative of chromosome 22 produced by translocation is passed on alongside the normal chromosome set, the zygote is viable. Unfortunately, such children of balanced carriers suffer from Emanuel syndrome which is characterized by a range of psychical disorders involving heart and kidney function as well as mental retardation [98].

Since the de novo translocations between PATRR11 and PATRR22 occur recurrently at similar breakpoints, Kurahashi et al. [99] were able to design a translocation-specific PCR assay to investigate the origins of translocation. The PCR assay at the single-molecule detection level was performed on sperm of men with a normal karyotype (46+XY). Approximately 1 in 10,000 DNA aliquots were positive for the translocation t(11;22) PCR product, indicating de novo translocation in sperm [100]. However, using the same methodology, the authors found no evidence of de novo t(11;22) translocation in blood and cheek swab cells from the same men, nor in lymphoblastoid cell lines and cultured fibroblasts. This points to the conclusion that de novo t(11;22) translocations occur during gametogenesis. Moreover, although a too small number of female oocytes are available to perform similar testing, there was some indication that occurrence of those de novo t(11;22) translocations might be sperm-specific. Ohye et al. [101] analyzed eight cases of offspring carrying derivative chromosome 22 and their parents. Due to the polymorphism between PATRR sequences originating from the mother and father, they were able to determine that in all eight cases, the de novo t(11;22) translocation was of paternal origin. The authors proposed that sperm specificity does exist, and it could be explained by a translocation occurring due to the palindrome-mediated instability during DNA replication. Namely, there is a great difference in the number of cell divisions (and thus DNA replications) in pre-meiotic gametogenic cells in males and females—around 150 divisions until adulthood and an additional 23 each year in spermatogenesis vs. 22 divisions throughout the female lifetime in oogenesis. However, although the frequency of mutations caused by replication errors as well as the frequency of some non-recurrent translocations in sperm does increase with age in men [102,103,104], this is not the case for de novo t(11;22) translocation [105].

In another line of research, the same research group demonstrated that, if there is sufficient negative superhelicity of DNA, PATRRs can extrude into a cruciform when cloned on non-replicative plasmids in human cells [106,107]. Further, the most recent experimental evidence by Correl-Tash et al. [108] demonstrated that PATRRs can extrude into a cruciform, leading to a higher frequency of DSBs in both mitotic and meiotic cells. They used sister chromatid exchanges (SCEs) observed in the metaphase chromosome spread using a florescent microscope in samples where PATRR regions were labeled with florescent probes. Since SCEs indicate sites at which DSBs occur and are repaired, they can be used as an indirect measure of genetic instability [109,110]. In mitotic cells (lymphocytes) of normal individuals, SCEs colocalize with PATRRs significantly more often than with a control region of the genome. Moreover, when DNA associated with DSB repair proteins (Rad51, NBS1 and γH2AX) was pooled by chromatin immunoprecipitation (ChIP), the qPCR analysis showed an increase in PATRR sequences in relation to various other control DNA regions. In both experiments, when DNA from balanced t(11;22) carriers was analyzed, no increase in SCEs frequency or DSB repair protein coprecipitation was detected. This is in accordance with the fact that after reciprocal translocation, the recombined PATRR sequences are no longer palindromic and thus no longer pose a threat to genome stability. The same ChIP-qPCR assay but using the cruciform-binding 2D3 antibody demonstrated an increased association between PATRRs and cruciform structures. Moreover, using the same methodology, they showed that PATRRs can result in cruciform formation, leading to an increase in DSB frequency and subsequent repair in spermatogenic cells, but this increase is not higher than in mitotic cells. Although it appears that the likelihood of PATRR-related cruciform extrusion and DSB formation is equal in mitotic and meiotic cells, a de novo PATRR-mediated translocation as a result of DSB repair is typically not found in mitotic cells [95]. Exceptionally, a case report described a woman with a mosaic karyotype, with 64% of normal cells and 36% of cells with t(11;22), indicating that translocation occurred in a post-zygote mitotic cell [111]. The explanation for most de novo PATRR-mediated translocation occurrences possibly lies in some specific DSB response or repair mechanism during gametogenesis.

Moreover, using the immunofluorescence labeling method and 2D3 cruciform binding antibody, Feng et al. [112] demonstrated the presence of cruciforms in mice growing oocytes. Interestingly, cruciform foci were detected in different phases of oocyte growth but were no longer present in fully grown oocytes (characterized by silencing of transcription activities, chromatin condensation and formation of a Hoechst-positive ring-like structure surrounding the nucleolus). Cruciform foci colocalized with PARP1 (poly(ADP-ribose)-polymerase, a protein involved in DNA damage sensing (mainly of single-stranded breaks), which was previously shown to bind hairpin loops [113], but not with γH2A.X, indicating that in normal oocytes, a cruciform-induced DSB is probably rare. Additionally, Feng et al. [112] analyzed data from The Genotype-Tissue Expression Project (GTEx, [114]) to see the expression pattern for PATRR11- and PATRR22-related transcripts in various human tissues. They found that the nearest transcript to PATRR11 (located about 1 kb downstream of PATRR11) is specifically expressed in liver and testes and that PATRR22 is located in the intron of a gene also specifically expressed in testes. Since DNA transcription induces a significant amount of negative superhelicity, they proposed that the transcription might be a driving process for cruciform extrusion. To further strengthen this hypothesis, they treated a transcriptional active growing oocyte with RNA-polymerase II inhibitor α-amanitin and noted a sharp decrease in the number of cruciform foci in treated cells.

Although palindromes PATRR11 and PATRR22 are the longest and thus the most recombinogenic, as well as the most extensively studied, the human genome harbors a dozen PATRRs. Palindromes PATRR8 (approximately 350 bp long) and PATRR17 (approximately 200 bp long), located on chromosomes 8 and 17, respectively, are also involved in constitutional translocations with PATRR22 as a partner. PATRR17 is located in the intron of the NF1 gene, and a t(17;22) translocation which led to inactivation of the NF1 gene was found in several patients with neurofibromatosis type 1 [115,116]. Recurrent t(8;22) translocation and inheritance of supernumerary derivative chromosome der(22)t(8;22) are linked to a syndrome characterized by ear and extremity abnormalities, in addition to mild mental retardation [117]. A translocation event between PATRR8 and PATRR11 has also been detected in sperm of healthy males [117]. Furthermore, the constitutional rearrangement between PATRR3 and PATRR8, t(3;8)(p14.2;q24.1), was shown to segregate with renal cell carcinoma in two families. Both PATRR8 and PATRR3 are located within introns of genes [118]. Non-recurrent translocations involving PATRRs on chromosomes 4, 1, 3 and 9 were also detected [119,120,121,122].

Additionally, there are examples where investigation of underlying molecular causes of various conditions in humans leads to the discovery of a palindrome in the genome clearly capable of instigating DNA rearrangements. In three unrelated families in Mexico and China, congenital generalized hypertrichosis (excessive hair growth all over the body) was linked to insertions mediated by a 180 bp palindrome located on chromosome X [123,124]. Likewise, a 160 bp palindrome located 30 kb upstream of the β-globin gene has been implicated in deletions leading to (ϵγδβ)^0^-thalassemia [125,126].

## 5. Concluding Remarks

Throughout this review, we tried to summarize the current state of knowledge on various aspects and consequences of compromised genome stability due to the presence of recombinogenic palindromic sequences. Although occasionally it appears that the research in this filed progresses more slowly than in some other areas due to the difficulties in the cloning and sequencing of DNA palindromes, recent advancements in our understanding of palindrome-instigated genome instability described in this review show that this subject holds the interest of researchers. Future research in this field will, on the one hand, continue to be focused on molecular mechanisms and protein players involved in palindrome recombinogenicity. It is important to understand when, why and under which conditions a certain palindromic sequence, which was stably replicated and inherited through numerous cell divisions, initiates genetic recombination, possibly with devastating consequences for the cell and organism. When it comes to cancer cells, genetic rearrangements are often abundant and complex, so it can be difficult to unravel the initiating and subsequent chain of events leading to a certain genotype. Undoubtedly, palindromic gene amplifications are an important mechanism involved in carcinogenesis. However, palindromes and inverted repeats preexisting in the genome could have a much greater role in initiating recurrent recombination events during carcinogenesis than is currently appreciated. Therefore, it is important to continue the deep sequencing efforts to fill the gaps in the human genome sequence which likely harbor multiple recombinogenic palindromic sequences, but also to analyze as much individual cancer genomes as possible and link them to specific cancer pathophysiology and treatment outcomes. Hopefully, with enough data analyzed, patterns will emerge which can improve diagnostics as well as the choice of appropriate treatment, but perhaps which can also uncover markers signifying elevated risk even before the disease onset.

## Abbreviations

BAC	bacterial artificial chromosome
BFB	breakage-fusion-bridge
DSB	double-strand break
GAPF	genome-wide analysis of palindrome formation
GWAS	genome-wide association study
NHEJ	non-homologous end joining
PATRR	palindromic AT-rich repeat
SINE	short interspersed element
SNP	single nucleotide polymorphism
TCGA	The Cancer Genome Atlas
